# An Unusual Case of Urachal Cyst Misdiagnosed as a Paraovarian Cyst: Ultrasound Assessment and Differential Diagnosis

**DOI:** 10.3390/diagnostics12123166

**Published:** 2022-12-14

**Authors:** Ciprian Ilea, Ovidiu-Dumitru Ilie, Irina-Liviana Stoian, Ioana-Sadyie Scripcariu, Bogdan Doroftei

**Affiliations:** 1 Faculty of Medicine, University of Medicine and Pharmacy “Grigore T. Popa”, University Street, No. 16, 700115 Iasi, Romania; 2 Clinical Hospital of Obstetrics and Gynecology “Cuza Voda”, Cuza Voda Street, No. 34, 700038 Iasi, Romania; 3 Department of Biology, Faculty of Biology, “Alexandru Ioan Cuza” University, Carol I Avenue, No. 20A, 700505 Iasi, Romania; 4 Origyn Fertility Center, Palace Street, No. 3C, 700032 Iasi, Romania

**Keywords:** hypogastric pain, urachal mucinous cystadenoma, ultrasound, magnetic resonance imaging, partial cystectomy, tumorectomy

## Abstract

The urachus is an embryologic remnant of the cloaca that usually degenerates after birth, resulting from the obliteration of the allantois, whose role is to connect the bladder to the umbilicus. Incomplete removal of the lumen may give rise to different malformations of the median umbilical ligament after birth. Although in the pediatric population urachus are common, most cases are asymptomatic and may go unrecognized until adulthood and give rise to cysts, rarely reported in the literature. Thus, in this manuscript we present the circumstances of a 43-year-old Romanian woman showing hypogastric pain of moderate intensity for three weeks, radiation in the left lower limb, menstrual cycle abnormalities, and dysmenorrhea. Based on the initial examinations, a paraovarian cyst measuring 80 mm was noted. Through the subsequent magnetic resonance imaging (MRI) conducted, a hypoechoic mass was detected, and the patient underwent a tumorectomy and partial cystectomy. A 9.7/7.5-cm tumor was excised, and the anatomopathological result was urachal mucinous cystadenoma. It came to our attention that relatively scarce data were found in the literature, with only seven studies with the diagnosis of the urachal cyst.

## 1. Introduction

The urachus is a three-layered allantois-derived embryologic remnant that develops before the fifth month of fetal development. Taking the shape of a fibromuscular tubular structure that descends into the pelvis, created through lumen obliteration, it degenerates after birth [1].

The primary role is to connect the dome apex with the umbilicus, compartmentalized by umbilico-vesical fascia along the medial umbilical ligament [2]. Postnatally, it extends and lies in the extraperitoneal cave of Retzius between the parietal peritoneum and the anterior abdominal wall [3].

However, there are occasions when this mechanism fails, which is not an uncommon phenomenon, and the epithelialized duct may persist until adulthood. The first documented primary urachal cancer (UC) was described in 1863 by Hue and Jacquin, responsible for around 0.5% and 20–40% of bladder malignancies and adenocarcinomas [4,5,6].

Thus, urachal anomalies (UA) caused by partial or incomplete obliteration could give rise to five distinct malformations. According to the latest figures, among the common UA forms stand congenital patent urachus (47%), urachal cyst (30%), umbilical-urachal sinus (18%), vesicourachal diverticulum (3%), and alternating sinus [7,8,9,10], aspects extensively already treated by Wilson et al. [2].

Although the urachal cysts incidence is one in 5000–150,000 in the adult and pediatric population [11,12], with a ratio of 2:1 in males than the older counterparts, it occurs in 1.6% of children under the age of 15 and 0.063% of cases in adults [13].

Unfortunately, due to infections and malignancy processes, if they do not resolve in the first two years, this might be the transition phase for the patients to become surgical candidates. In the absence of complications, anomalies are found incidentally for other medical reasons [14,15].

On the other hand, 35% of patients may present to the Emergency Department reporting lower abdominal pain [16], signs of urinary tract infection, painful abdominal lump, or hematuria. Therefore, the emphasis might be orientated upon this clinical panel since these symptoms mimic the spectrum resembling Meckel’s diverticulum, appendicitis, or incarcerated hernia [17].

Thus, this manuscript presents the case of a Romanian female patient with a urachal mucinous cystadenoma initially misdiagnosed with a paraovarian cyst.

## 2. Case Presentation

### 2.1. Patient Information

A 43-year-old Romanian female patient (M.P.) presented to the “Cuza Voda” Obstetrics and Gynecology Clinical Hospital from Iasi, reporting hypogastric pain of moderate intensity whose onset started three weeks prior, with radiation in the left lower limb and accompanied by menstrual cycle abnormalities and dysmenorrhea.

### 2.2. Clinical History

She declared having irregular scanty flow during menstruation, and dysmenorrhea, with two previous Caesarean (C)-sections. Moreover, she suffered a cholecystectomy complicated by a septic condition, but her bladder and bowel habits were normal.

### 2.3. Physical Examination and Paraclinical Tests

During the standard evaluation, an enlarged abdomen in volume was observed due to the adipose panicle, sensitive to palpation in the hypogastrium and Pfannestiel-associated scars following the C-sections and cholecystectomy. She did not complain of urinary symptoms, such as dysuria, pollakiuria, hematuria, or micturition disorders.

Furthermore, the laboratory test results indicated anemia, with the value of the tumoral antigen CA125 of 132.3 U/mL (0–35 U/mL) and human epididymis secretory protein (HE) of 28.9 pmol/mL (<70 pmol/mL), indicating a risk of ovarian malignancy algorithm (ROMA) of 7.4% (<7.4%), also with the presence of calcium oxalate crystals in the urine exam.

### 2.4. Diagnostic Assessment and Investigations

**Figure 1:**
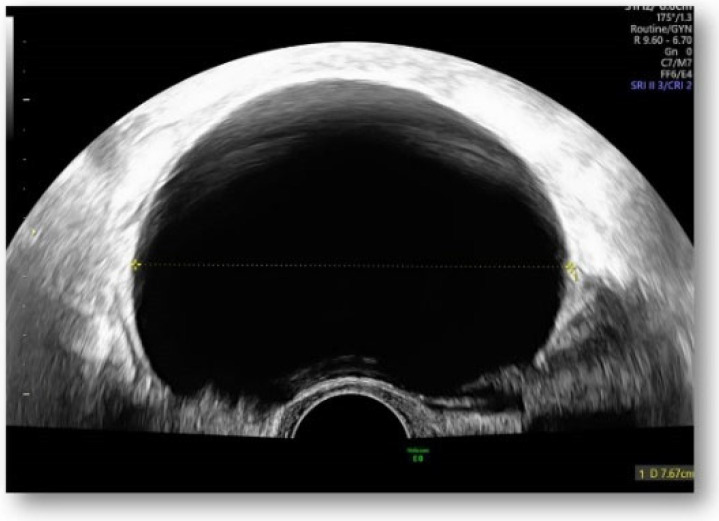
Large hypoechoic cystic formation located to the left of the uterus measuring 80 mm.

**Figure 2:**
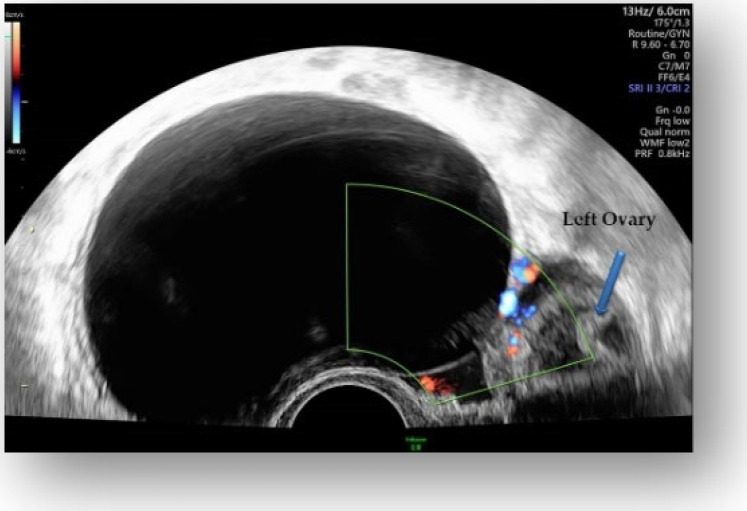
Hypoechoic cystic formation with peripheral doppler flow, without intracystic vegetations.

**Figure 3:**
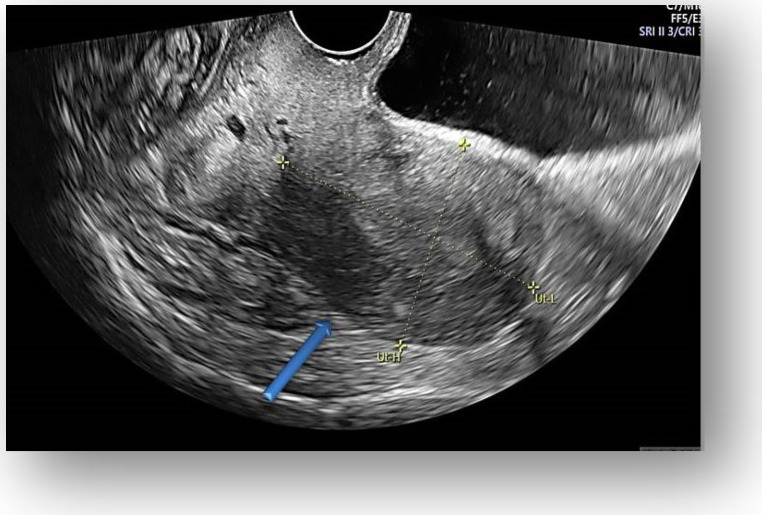
Transvaginal ultrasound showed uterine body with normal endometrium.

**Figure 4:**
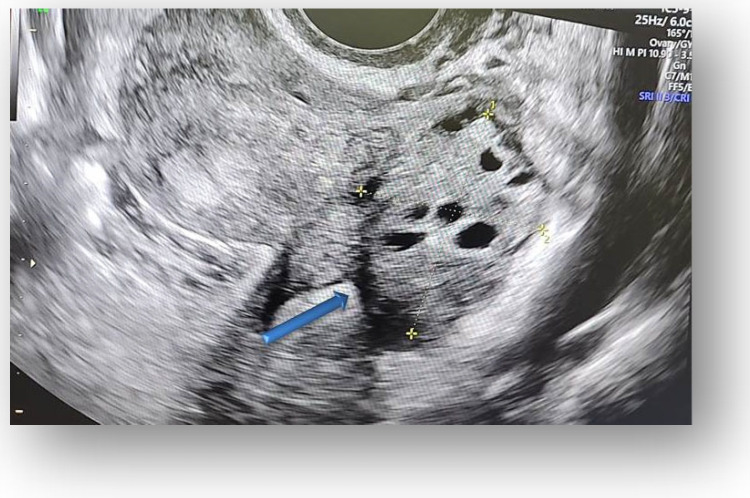
The contralateral ovary has a normal appearance and size.

Considering all the information gathered, we first decided to perform an ultrasound (US) which revealed a large hypoechoic cystic formation on the left of the uterus, misdiagnosed as a paraovarian cyst measuring 80 mm (Figure 1, Figure 2, Figure 3 and Figure 4).

The subsequent MRI revealed slight motion artifacts due to intestinal peristalsis.

The bladder had homogeneous content, without intraluminal protruding formations; imprinting of the medial and left-sided bladder dome by the dominant left adnexal lesion.

The uterus located paramedian right was enlarged, with global dimensions of approximately 145 mm longitudinal/75 mm sagittal/90 mm transverse, and the endometrium had ~6 mm thick with no particularities. There was a diffuse thickening of the junctional area of the anterior uterine wall, up to ~42 mm, suggestive of adenomyosis. The junctional site thickness of the posterior wall was within normal limits (up to ~8 mm). The elongated cervix, up to ~45 mm, did not present macroscopic suspicious tumor lesions evident on magnetic resonance (MR).

The right ureter was slightly ectatized posteriorly and cranially by the uterine fundal region (diameter 4–7 mm), probably by a degree of compression by the enlarged uterus. The upper pole of the uterus was located approximately next to the lower vertebral plateau L4.

The uterus came posterior to the right ureter, the right common iliac vascular bundle, and its bifurcation. Anteriorly, it came into contact with the anterior pelvic wall (right rectus abdominis muscle, inferior epigastric vascular bundle), laterally with the right external iliac vascular bundle, medially with the described cyst, infero-medially with the urinary bladder, and cranially with intestinal loops.

The right ovary, with a long axis of about 30 mm, was located right parauterine, ascending in the right iliac fossa, immediately caudal to the ileo-cecal region, showing some infracentimetric follicular images.

The left ovary, with the long axis of about 32 mm, was located between the anterior pelvic wall and the left external iliac vascular bundle, about 7 cm lateral to the midline showing some follicular (possibly cystic) images up to ~16 mm.

**Figure 5:**
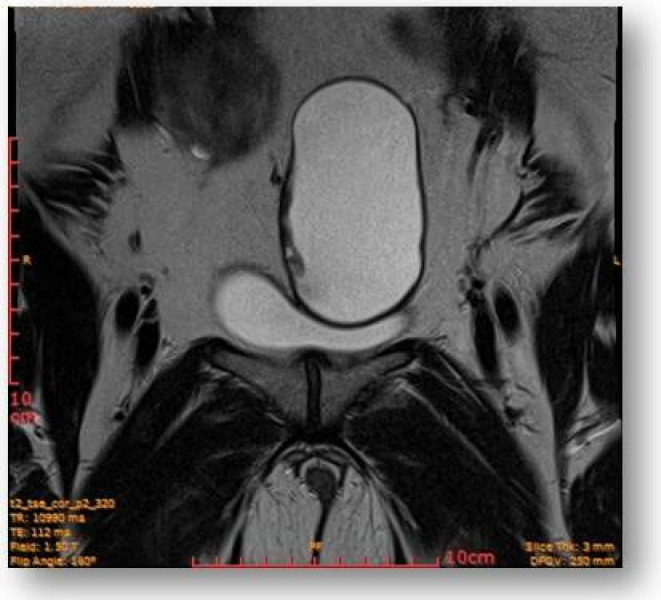
Left parauterine, between the left lateruterine wall and the left ovary attached to the anterior outline of the round ligament, an ovoid cystic lesion of 100/68/50 mm is evident.

Left parauterine, between the left lateruterine wall and the left ovary, was attached to the anterior contour of the uterine round ligament, and an ovoid cystic lesion of approximately 100/68/50 mm (oblique CC/AP/LL) is evident (Figure 5).

**Figure 6:**
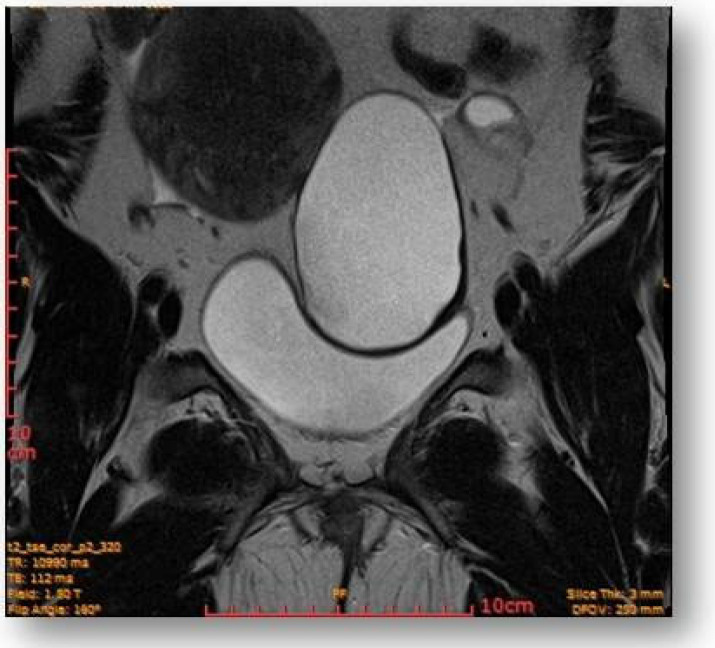
Ovoid cystic lesion imprints the urinary bladder without invasion into it.

This showed probably proteinaceous fluid content and mildly irregular wall thickness up to ~4–5 mm (on the right lateral contour) and with a mural micronodule of ~5 mm with contrast uptake at the level of the left antero-lateral contour. The appearance advocated the first hypothesis for a “border-line” left paraovarian cyst, with suspicious elements of neoplastic transformation (Figure 6). It presented the following relationships: anteriorly, it imprints the anterior median-paramedian left pelvic wall, posteriorly with the uterine round ligament, urinary bladder, medially (to the right) with the uterus and urinary bladder, laterally (to the left) with the left ovary.

**Figure 7:**
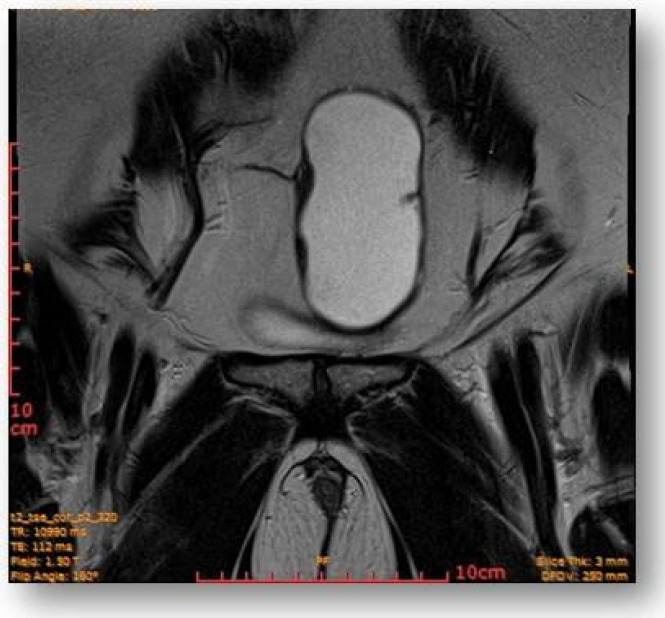
Ovoid cystic lesion proteinaceous fluid content and mildly irregular wall thickness up to ~4–5 mm.

**Figure 8:**
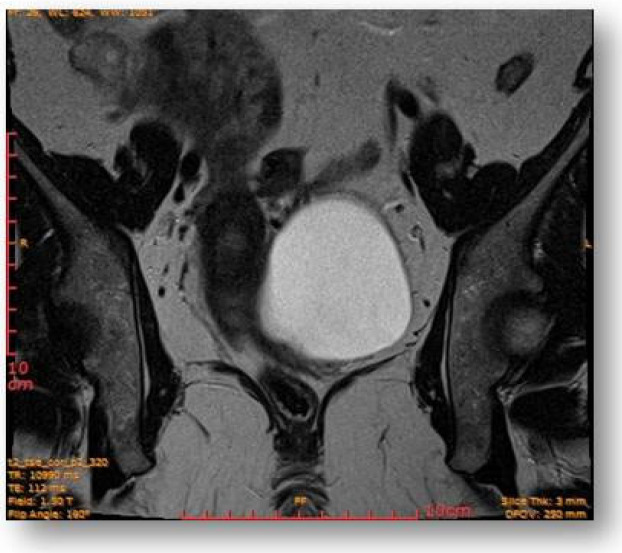
Ovoid cystic lesion in the left part of the uterus.

The rectal ampulla did not show suspicious tumor masses. Several infracentimetric pelvic lymph nodes, without specific character, were noted, as well as a minimal plate of intrapelvic fluid with a millimeter thickness. No notable collections in the pelvic recesses. No suspicious focal bone lesions were evident in the pelvis on the acquired images. Diastasis of the rectus abdominis muscles in the pelvic region (distance of about 30–35 mm) could be observed (Figure 7 and Figure 8).

### 2.5. Surgical Treatment

**Figure 9:**
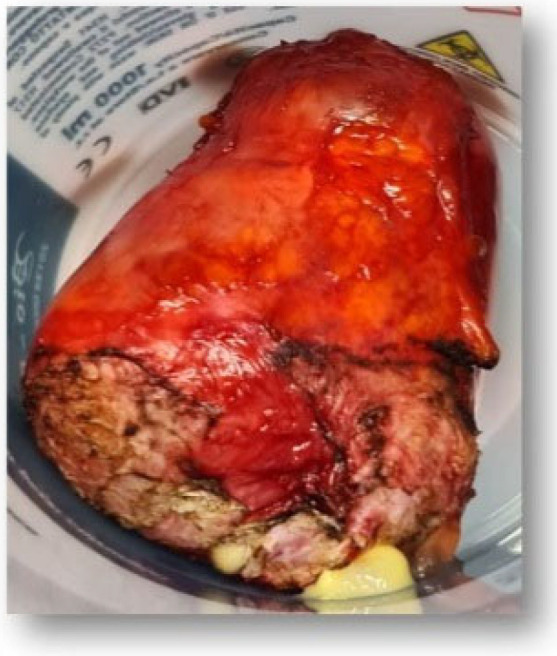
Anatomopathological specimen.

We decided to recommend a laparoscopy, but unfortunately, the patient refused this approach accusing the unfavorable evolution of a previous laparoscopic cholecystectomy. Thus, we performed a surgical intervention via laparotomy, and a tumor formation of 9 cm adherent to the anterior abdominal wall was identified (Figure 9). After the adhesiolysis, the internal genital organs were visible, and the uterus and ovaries appeared normal; the tumor was adherent to the posterior bladder wall. A urologist was requested, and tumorectomy and partial cystectomy were conducted, the benign cyst being subjected to paraffin embedding for the extemporaneous anatomopathological examination. However, we were unable to establish the origin of the tumor. There were no complications post-intervention, and the patient had a healthy follow-up.

### 2.6. Anatomopathological Result: Urachal Mucinous Cystadenoma

*Macroscopic examination*: (1) There was a tumor formation of 9.7/7.5 cm, with a small lack of substance on the cystic section, unilocular, with gelatinous, viscous content, thin wall, without internal vegetations. (2) Bladder wall fragment was 4/1/0.8 cm.

*Microscopic examination*: (1) Cyst walls represented by conjunctive-muscular tissue and adherent adipose tissue, wallpapered only focally on a slope of cylindrical unistratified epithelium with apical mucosecretion, without atypia, next to extended beaches of mucus in which multinucleated giant cells and inflammatory cells were observed, floating epithelial cells. Intramurally, focal calcifications, and mucus focally dissecting the cyst wall were noted. (2) Bladder wall fragments represented by connective-muscular tissue, with notable congestion and chronic inflammation were evident.

## 3. Discussion

The urachus, known as the median umbilical ligament, is a midline fibrous remnant structure of the cloaca via the obliteration of the allantois that extends cranially to the umbilicus and degenerates after birth, laying between the peritoneum and the transversalis fascia [18], the primordial abnormality being described by Cabriolus in 1550 [19].

Thus, the cysts are vestigial remnants that can lead to complications, particularly affecting the males, and can be asymptomatic until adulthood upon becoming infected, including bladder fistula formation, cyst rupture, peritonitis, and sepsis, caused by *Escherichia coli*, *Enterococcus faecium*, *Klebsiella pneumonia, Proteus, Streptococcus viridans,* and *Fusobacterium* [20,21]. Besides the plethora of symptoms, it may be characterized by local periumbilical or lower abdominal pain, urinary manifestations, fever, omphalitis, and pain when the mass is palpable [22,23,24].

There are publications concerning urachal cysts, but those referring to malformations of 7 cm are relatively limited. Therefore, we found it appropriate to summarize the main aspects that are tangent with our case report in Table 1.

Ashley et al. [22] conducted a longitudinal study from 1951 to 2005 in which they retrieved the medical records of *n* = 176 patients aiming to associate the clinicopathological findings with malignancy in adults through univariate and multivariate logistic regression models. They identified urachal remnants in *n* = 46 children and *n* = 130 adults, from which *n* = 46, 43% (*n* = 20) presented with umbilical drainage, and 50% (*n* = 23) were diagnosed by physical examination and *n* = 34 children, 74% underwent a simple excision. Concerning the adults, 50% (*n* = 65) exhibited hematuria, 60% (*n* = 78) needed cystoscopy, and 41% (*n* = 53) CT, but 51% (*n* = 66) involved comprehensive operation, such as partial or radical cystectomy. Cumulatively, 51% (*n* = 66) were malignant, older patients (≥55-years-old) suffering from hematuria classified as strong predictors (*p* < 0.001), once with aging.

Subsequently, Chiarenza and Bleve [31] performed a retrospective study over a decade (2006–2016) which included *n* = 16 children diagnosed with urachal anomalies having abdominal or urinary symptoms, *n* = 8 being subjected to an open excision (5.5 years average—4 months–13 years-old), and *n* = 8 by laparoscopic surgery (10 years average—1 month–18 years-old). In this manner, the authors showed a mean operative time of 63 min (35–105 min) and 50 min (35–90 min) in both groups with no postoperative complications, discharge interval between 48 and 96 h, and a benign urachal remnant in each case.

Considering all the aspects mentioned above, we performed further searches in the literature and even fewer entries were returned with the result of urachal mucinous cystadenoma depending on the age of the patient, size, and histology.

The diagnostic criteria panel has undergone revisions throughout the years, and the accepted ones are those according to Hamilou et al. [32] and described by Sheldon, Mostofi et al. [33,34] in 1984 and 1955. Unfortunately, a uniform, standardized, and optimal protocol for the management approach of urachal anomalies remains controversial and under debate, mainly depending on variables, such as the patient’s age and the severity of the condition [3,35].

Despite the discrepancies in the current literature concerning this matter, there is pinpointed an increased interest in the prophylactic surgical removal of asymptomatic urachal remnants. Even though experts in the field advocate surgical interventions in both pediatric and adult groups whether they are (a)symptomatic, there are also situations when patients with non-specific atretic urachal remnant and specific symptomatic patients are be managed non-operatively [36,37]. In any of these scenarios, US at follow-up is recommended to exclude any possible remnant in these patients [24].

Some authors support the removal of non-infected urachal remnants as an alternative to avoid future emergency surgery because it carries a higher risk such as infection and malignancy [36]. In contrast, others argue for the potential of urachal remnants’ malignant tissue degeneration and causing the formation of neoplasms, which is essential, especially in those with a history of malignant transformation [9,38]. However, it would be an aggressive procedure in children, and the possibility is very rare [18].

Continuing with this concept, some advocate for a two-stage management technique involving the usage of antibiotics and US-guided drainage prior to the operative excision for infections [39,40,41], whereas others support the removal and do to not attribute an associated interval for infection and inflammation to reduce [23,39].

Nevertheless, it should be emphasized that most asymptomatic cases in earlier stages of development may further reflect in diagnostic delays and poor prognosis, with it even being theorized that a neglected situation might become silent and culminate in infections, chronic inflammation with an increased risk for carcinogenesis [22], recurrent urinary tract infections, and stone formation [9].

Imaging techniques, such as computed tomography, MRI, and ultrasonography (USG), constitute the radiologic core method of choice and play a pivotal role in differentiating the urachal cysts from other causes [32,42], providing information about the size and relationship with the peripheral tissue [11,12,23,35,43,44,45].

## 4. Conclusions

The peculiarity of the case lies in the rarity of the pathology, although it is not malignant. To the authors’ best knowledge, this is the only one reported in our country and in the northeastern region of Europe. The pathology is still difficult to diagnose preoperatively. A non-concordant imaging diagnosis, as in the presented case, can lead to incorrect management and affect the prognosis, especially in monodisciplinary hospitals, with limited resources, requiring a multidisciplinary team. The case draws attention to this pathology and the importance of a correct imaging diagnosis, especially in areas with limited resources, in order to direct the patient to a specialized center.

## Figures and Tables

**Table 1:** Overview of case reports reporting urachal cysts over 7 cm.

Age	Sex	Symptoms	Size (cm)	Treatment	Histology	Reference
Thirty-five	Male	Dysuria, perineal pain	11.5 × 6 × 3	Partial cystectomy *	Squamous cell carcinoma of the urachus	[25]
Thirty-eight	Female	Lower abdominal pain	14 × 7 × 6.5	Laparoscopy *	Tubo-ovarian abscess and Cystic urachal remnant	[15]
Forty-three	Female	Lower abdominal pain	9.7 × 7.5	Partial cystectomy and Tumorectomy	Urachal Mucinous Cystadenoma	Present case
Fifty-nine	Male	Hematuria, lower abdominal pain	7 × 4	Partial cystectomy *	Squamous cell carcinoma of the urachus	[26]
Sixty-two	Female	Suprapubic pain	16 × 14 × 11	Radiotherapy and Chemotherapy	Squamous cell carcinoma of the urachus	[27]
Sixty-four	Male	Dysuria, lower abdominal pain	9 × 6	Partial cystectomy *	Squamous cell carcinoma of the urachus	[28]
Seventy	Male	Dysuria, hematuria	8 × 6 × 6	Partial cystectomy *	Squamous cell carcinoma of the urachus	[29]
Eighty-four	Male	Dysuria, lower abdominal pain	8 × 7 × 6	Partial cystectomy *	Squamous cell carcinoma of the urachus	[30]

*—en bloc.

## Data Availability

The datasets used and analyzed in this study are available from the corresponding author on reasonable request.
